# Association of the *ACTN3* R577X Polymorphism in Polish Power-Orientated Athletes

**DOI:** 10.2478/v10078-011-0022-0

**Published:** 2011-07-04

**Authors:** Paweł Cięszczyk, Jerzy Eider, Magdalena Ostanek, Aleksandra Arczewska, Agata Leońska-Duniec, Stanisław Sawczyn, Krzysztof Ficek, Krzysztof Krupecki

**Affiliations:** 1 University of Szczecin, Department of Physical Culture and Health Promotion, Poland; 2 University of Edynburg, Department of Genetics, England; 3 Pomeranian Medical University in Szczecin, Department of Clinical and Molecular Biochemistry, Poland; 4 University of Szczecin, Department of Genetics, Poland; 5 Academy of Physical Education and Sport, Department of Sport Education, Poland; 6 Galen Medical Center, Poland

**Keywords:** α-actinin-3, genotype, power-orientated athletes

## Abstract

Alpha-actinins are an ancient family of actin-binding proteins that play structural and regulatory roles in cytoskeletal organization. In skeletal muscle, α-actinin-3 protein is an important structural component of the Z disc, where it anchors actin thin filaments, helping to maintain the myofibrillar array. A common nonsense polymorphism in codon 577 of the ACTN3 gene (R577X) results in α-actinin-3 deficiency in XX homozygotes. Based on knowledge about the role of ACTN3 R557X polymorphism in skeletal muscle function, we postulated that the genetic polymorphism of ACTN3 could also improve sprint and power ability.

We compared genotypic and allelic frequencies of the ACTN3 R557X polymorphism in two groups of men of the same Caucasian descent: 158 power-orientated athletes and 254 volunteers not involved in competitive sport.

The genotype distribution in the group of power-oriented athletes showed significant differences (P=0.008) compared to controls. However, among the investigated subgroups of athletes, only the difference of ACTN3 R577X genotype between sprinters and controls reached statistical significance (P=0.041). The frequencies of the ACTN3 577X allele (30.69% vs. 40.35%; P=0.005) were significantly different in all athletes compared to controls.

Our results support the hypothesis that the ACTN3 577XX allele may have some beneficial effect on sprint-power performance, because the ACTN3 XX genotype is significantly reduced in Polish power-oriented athletes compared to controls. This finding seems to be in agreement with previously reported case-control studies. However, ACTN3 polymorphism as a genetic marker for sport talent identification should be interpreted with great caution.

## Introduction

Twin studies indicate that muscle function phenotypes are highly heritable (Arden and Spector, 1997; Spurway and Wackerhage, 2006) and various candidate genes have been studied to explain some of the high inter-individual variability observed in these phenotypes, e.g. myostatin gene (*MSTN*), angiotensin converting enzyme gene (*ACE*), peroxisome proliferator-activated receptor-γ coactivator 1α (*PGC-1*α), hypoxia-inducible factor-1 (*HIF-1*) and others (Buchner et al., 1993; Spurway and Wackerhage, 2006). Therefore, only a small number of genes containing single nucleotide polymorphisms (SNPs) have been identified as candidate genes for explaining this variability (Delmonico et al., 2008). One of them is alpha-actinin-3 gene (*ACTN3*), which encodes the protein a-actinin-3.

Alpha-actinins are an ancient family of actin-binding proteins (Mills et al., 2001) that play structural and regulatory roles in cytoskeletal organization. In skeletal muscle, two alpha-actinin proteins (α-actinin-2 and α+actinin-3) are an important structural component of the Z disc, where they anchor actin thin filaments, helping to maintain the myofibrillar array (Beggs et al., 1992; Blanchard et al., 1989). Besides their mechanical role, both sarcomeric alpha-actinins interact with proteins involved in numerous signalling and metabolic pathways (MacArthur and North, 2004; Mills at al., 2001). While α-actinin-2 is expressed in all types of muscle fibers, the expression of α-actinin-3 is almost exclusively restricted to fast, glycolytic type II fibers (Clarkson et al., 2005).

In 1999, North et al. (1999) identified a common polymorphism in *ACTN3* R577X (dbSNP rs1815739) that results in absence of α-actinin-3 in more than one billion people worldwide, despite the *ACTN3* gene being highly conserved during human evolution. Though this genetic variation is not associated with any known disease phenotype, the α-actinin-3-deficient XX genotype is believed to preclude top-level athletic performance in ‘pure’ power and sprint sports (North et al., 1999; Suminaga et al., 2000; Druzhevskaya et al., 2008).

The first evidence that a mononucleotide difference in DNA sequence was associated with power ability referred to the R577X polymorphism of the *ACTN3* gene was indicated by Yang et al. (2003). The translation (C>T) at nucleotide position 1747 in the ACTN3 coding sequence converts an arginine (R) to a stop codon (X) at residue 577 (Mills et al., 2001). This variation creates two different versions of the *ACTN3* gene, both of which are common in the general population: the 577R allele is the normal, functional version of the gene, whereas the 577X allele contains a sequence change that prevents completely production of functional α-actinin-3 protein (Ahmetov et al., 2008).

Till this moment, many reports suggested that the presence of *ACTN3* may have a beneficial effect on skeletal muscle function (Niemi and Majamaa, 2005; Papadimitriou et al., 2007; Roth et al., 2008; Santiago et al., 2008), especially in the case of generating powerful contractions at high velocity (McCauley et al., 2009). As was mentioned above, the α-actinin-3 is a part of the sarcomeric α-actinins, which are major components of the Z line, where its function is twofold: to connect with actin filaments and sustain the order of myofilaments and coordinate myofilament contraction (Yang et al., 2003). The Z line is an important structure within the sarcomere and its function is to provide structural support for the transmission of force when the muscle fibres are activated (Wilmore et al., 2004). It is suspected that α-actinin-3 may be optimized to decrease the damage induced by eccentric muscular contraction (Yang et al., 2003).

Additionally, same reports indicated the positive association between the presence of the 577R allele and the capacity to perform high power muscle contractions (Clarson et al., 2005; Delmonico et al., 2007). Furthermore, Vincent et al. (2007) showed that the percentage surface and number of type IIx fibers (fast-twitch glycolytic, which are used predominantly in highly explosive events, such as the 100-m sprint) was greater in the RR than the XX genotype of young healthy men.

Based on knowledge about the role of *ACTN3* R557X polymorphism in skeletal muscle function, we postulated that the genetic polymorphism of *ACTN3* could also improve sprint and power ability.

The aim of this study was to perform preliminary studies to analyze the possible importance of the *ACTN3* R577X polymorphism in Polish power-orientated athletes as well as in sedentary individuals representing possible relationships with the genotype and physical performance.

## Material and methods

### Ethics Committee

The Pomeranian Medical University Ethics Committee approved the study and written informed consent was obtained from each participant.

### Subjects and controls

158 male power-orientated athletes of regional or national level with no less than 8 years training experience were recruited for this study (48 sprinters, 54 sprint swimmers, 56 weightlifters).

As a control group, samples were prepared from 254 unrelated volunteers (male students from the University of Szczecin). The athletes and controls were all Caucasian to ensure there was no ethnicity skew and to overcome any potential problems of population stratification.

### Genotyping

The buccal cells donated by the subjects were collected in Resuspension Solution (Sigma, Germany) with use of Sterile Foam Tipped Applicators (Puritan, USA). DNA was extracted from the buccal cells using GenElute Mammalian Genomic DNA Miniprep Kit (Sigma, Germany) according to the producer protocol.

The 290 bp fragment of exon 15 of the ACTN3 gene was amplified by PCR using the forward primer : 5′-CTGTTGCCTGTGGTAAGTGGG-3′ and the reverse primer : 5′-TGGTCACAGTATGCAGGAGGG-3′ as recommended by Mills et al. (2001). PCR reaction mix (total volume 10 μl) contained 1.5 mM MgCl2, 0.75 nM of each deoxynucleoside triphosphate (Novazym, Poland), 4 pM of each primer (Genomed, Poland), 0.5 U of Taq DNA polymerase (Sigma, Germany), and 1 μl (30–50 ng) of template DNA. After a first step consisting of 95°C for 5 min., 35 cycles of amplification were performed by using denaturation at 95°C for 30 s, annealing at 60°C for 30 s, and elongation at 72°C for 30 s and a final cycle at 72°C for 10 min (Lucia et al., 2006). The amplified PCR fragments were subsequently digested with *Dde I* endonuclease (Fermentas, Lithuania) in a condition recommended by the supplier (Mills et al., 2001). The alleles 577R and 577X were distinguished by the presence (577X) or absence (577R) of a *Dde I* restriction site. Digestion of PCR products of the 577X allele yields bands of 108, 97 and 86 bp, whereas digestion of PCR products of the 577R allele yields bands of 205 and 86 bp. The digested products were separated by 3% agarose gel electrophoresis, stained with ethidium bromide, and visualized in UV light.

### Statistical analysis

Genotype distribution and allele frequencies between groups of athletes and controls were compared and significance was assessed by χ2 test using STATISTICA 8 statistical package. *P* values of < 0,05 were considered statistically significant.

## Results

*ACTN3* genotype distributions amongst subjects and controls were in Hardy-Weinberg equilibrium, making selection bias less likely. Genotype distribution results of the control group (RR-35,83%; RX-49,21%; XX-14,96%) were similar to those reported in previous studies on Caucasian populations (North et al., 1999; Yang et al., 2003; Ahmetov et al., 2008; Druzhevskaya et al., 2008). The distributions of the *ACTN3* genotypes and alleles are given in Table 1. Genotype distribution in a whole cohort of athletes showed significant difference (*P*=0.008) compared to controls. However, among the investigated subgroups of athletes, only the difference of *ACTN3* R577X genotype between sprinters and controls reached statistical significance (*P*=0.041)

**Figure 1:**
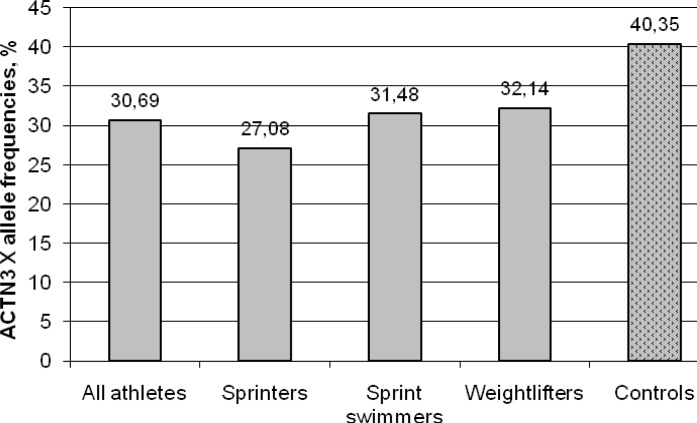
ACTN3 577X allele frequency amongst power-oriented athletes and controls is shown. X allele genotype frequency in controls was 40,35%. By comparison, it was 30,69% (P=0.005); 27.08% (P=0.014); 31.48% (P=0.085) and 32.14%% (P=0.106) for all athletes, sprinters, sprint swimmers and weightlifters.

The frequencies of the *ACTN3* 577X allele (30.69% vs. 40.35%; *P*=0.005) were significantly different in all athletes group compared to controls. This trend was worse when comparing each subgroups of investigated athletes to controls separately (Figure 1).

## Discussion

The first evidence for strong *ACTN3* gene influence on athletic performance was first reported by Yang et al. (2003). The possible mechanism underlying the association of the *ACTN3* R577X polymorphism with athletic performance was discussed in detail by MacArthur and North (2007) and MacArthur et al. (2007).

The presented report is the first to indicate that *ACTN3* XX genotype is under-represented in Polish strength-power orientated athletes in comparison with controls. Here, we also show that the distribution of *ACTN3* genotypes and alleles in Polish population is similar to these observed in several reported groups of Caucasian populations (Druzhevskaya et al., 2008; Ahmetov et al., 2008; Moran et al., 2007; North et al., 1999; Yang et al., 2003).

Our report suggests that the *ACTN3* RR and RX genotypes are associated with predisposition to power-sprint sports, because the *ACTN3* XX genotype is significantly reduced in Polish power-oriented athletes compared to controls. This finding seems to be in agreement with previously reported case-control studies.

The mentioned above report by Yang et al. (2003) indicated that only 6 % of elite Australian sprinters were homozygous for the 577X genotype. This finding seems to be supported by Druzhevskaya et al. (2003), who investigated 486 male and female Russian athletes of regional or national sports level. The mentioned study showed that variation in the *ACTN3* gene was strongly associated with elite power athlete status in Russians. Also Eynon et al. (2009) found that the proportion of subjects homozygous for the 577R allele was significantly higher in sprinters (50 %) compared to endurance athletes and controls (19 % and 20 % respectively). Moreover, 88 % of the top-level Israel sprinters had at least one copy of the 577R allele, indicating the crucial role of this allele in the development of top-level sprint ability.

The association between the RR genotype of *ACTN3* and elite athletic performance was also demonstrated in Finnish sprint athletes (Niemi and Majamaa, 2005). The comparison of endurance and sprint athletes has shown that the frequency of the XX genotype of *ACTN3* was lower and that of RR higher among sprinters than among endurance runners (*P*=0.03). Furthermore, none of the top sprinters harboured the XX genotype.

Another study suggested that the presence of α-actinin-3 protein had a positive effect on power performance. A report by Papadimitriou et al. (2008) showed statistically significant differences in the frequencies of alleles (*P*=0.017) and genotypes (*P*=0.016) between elite power-orientated athletes and the control group (the power orientated athletes displayed a lesser frequency of the 577X allele than the control group). It is worth to mention that all of the investigated Olympic/European- level sprinters had at least one 577R allele of *ACTN3*.

However, the role of the *ACTN3* R577X polymorphism in athletic performance has not yet been sufficiently clarified. Norman et al. (2009) indicated that R577X polymorphism in *ACTN3* is not associated with differences in power output, fatigability, or force-velocity characteristics in physically active individuals. Moreover, Norman et al. (2009) suggested that alpha-actinins do not play any significant role in determining muscle fiber-type composition. Additionally, McCauley et al. (2009) indicated that *ACTN3* R577X polymorphism did not influence absolute or relative torque at high velocities or the twitch response. This finding seems to be supported by Delmonico et al. (2008), who showed that in older women (64 years), knee extensor concentric peak power was found to be higher in 577X allele homozygotes compared with RR genotype individuals. Similarly, Clarkson et al. (2005) reported no association between *ACTN3* R577X genotype and muscle phenotype in men when investigating isometric elbow flexor strength. Finally, Lucia et al. (2007) reported a case of a Spanish elite long jumper (two times Olympian) whose genotype for the *ACTN3* gene is XX. The similar finding was made by Druzhevskaya et al. (2008), who observed one highly elite Russian hammer thrower (world record holder) with XX genotype.

Our study had some restrictions. The investigated group consisted mostly of regional or national level athletes. On the other hand, even in this group, the study proved that the *ACTN3* R577X allele could be one of the factors influencing power-oriented sport disciplines. Such findings have important implications for understanding of molecular mechanisms underlying the predisposition to high power potential and support the hypothesis that the presence of α-actinin-3 has a beneficial effect on the function of skeletal muscle in generating forceful contractions at high velocity (Druzheyskaya et al., 2008).

It is worth noting that among all reported subgroups (i.e. sprinters, swimmers and weightlifters) statistically significant differences in genotype distributions were only demonstrated in sprinters. This fact can be explained by the specificity of performed movements and big loads, resulting in a greater risk of injury in sprinters than in swimmers or weightlifters. Presumably, that is the reason why having at least one *ACTN3* R577 allele is a key factor in sprint training.

On the other hand, the differences in the genotype distribution between the subgroups were not statistically significant. This fact suggests that the *ACTN3* R577 allele may also have a beneficial role in swimmers and weightlifters. Unfortunately, the relatively small size of subgroups in this study can cause some ambiguity in the obtained results. For example, the frequency of allele *ACTN3* 577X in swimmers (54) was 31.48 % and was statistically insignificant (p= 0.085), although, it was only 0.79 % lower than the frequency of this allele in all the examined athletes (158) which proved to be highly statistically significant (p= 0.005)). Therefore, our conclusions should be supported with more experimental studies on *ACTN3* polymorphisms in elite athletes.

## Figures and Tables

**Table 1:** ACTN3 genotype distribution of the athletes and controls (data is presented as relative values

***Group***	***n***	***Genotypes, %***			***P value***
		***RR***	***RX***	***XX***	
All athletes	158	44,94	48,73	6,33	0,008^*^
Sprinters	48	50,00	45,83	4,17	0,041^*^
Sprint swimmers	54	42,59	51,85	5,56	*0,130*
Weightlifters	56	42,86	50,00	7,14	*0,205*
Control	254	35,04	49,21	15,75	*-*

* p<0.05 compared to control.

Comparison with controls was by χ2 test RR Wild-type homozygote, RX heterozygote, XX mutant homozygote
